# Supplementary material for: Diagnostics of preeclampsia based on Congo red binding to urinary components: Rationales and limitations
Source: PLoS One. 2024 Jan 19;19(1):e0297144. doi: 10.1371/journal.pone.0297144 (PMC10798433; doi:10.1371/journal.pone.0297144)
Supplement: S1 Raw images — (PDF) [file pone.0297144.s002.pdf]

Fig. 5. Congophilia of urine samples from patients of different groups before and after ultracentrifugation.

Samples were stained with Congo red, applied on nitrocellulose membrane, washed in ethanol and imaged with camera (Redmi Note 10, ISO 100) in lab-made box with two LED lamps.

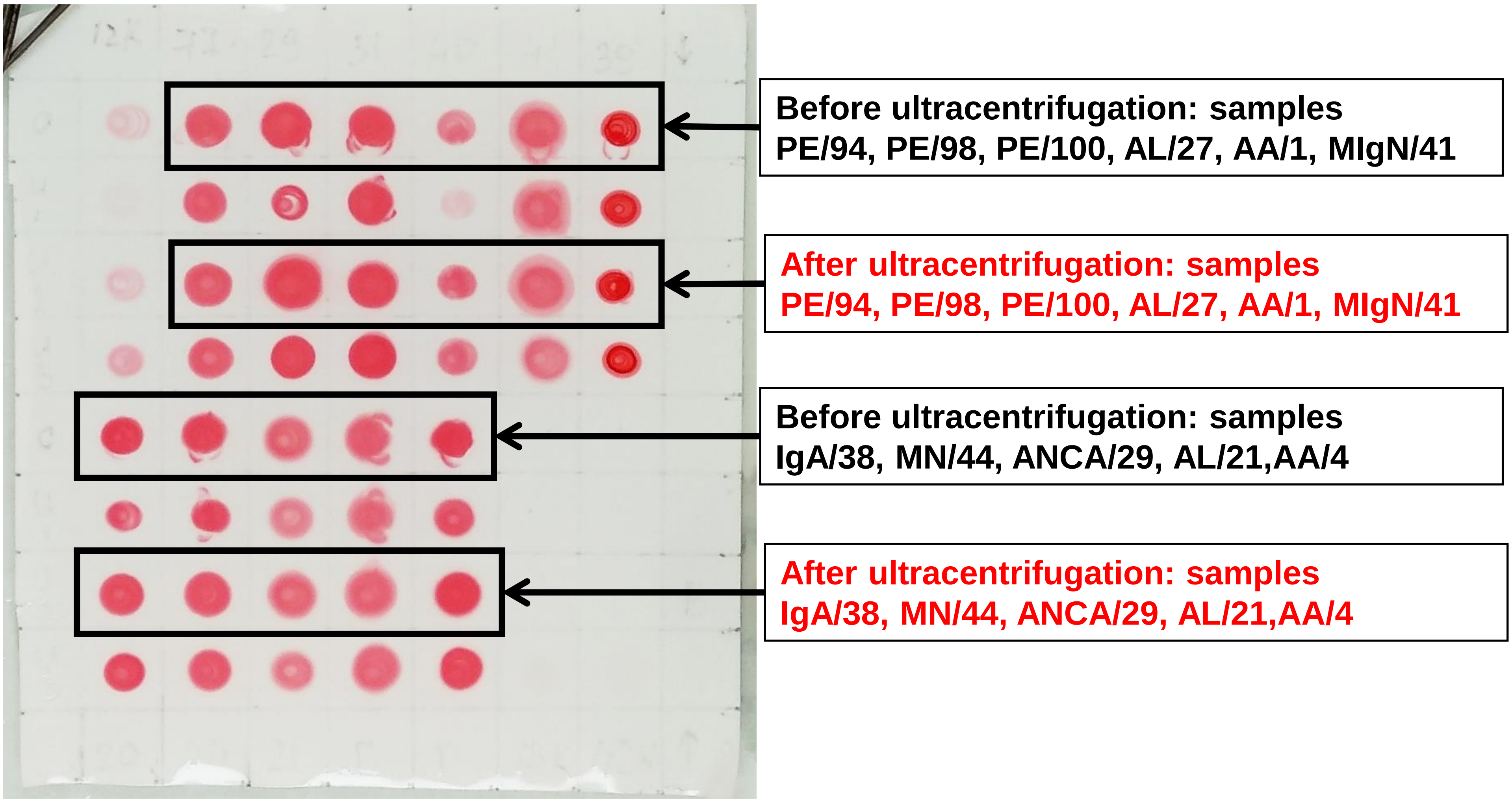

AA, serum amyloid A amyloidosis; AL, Immunoglobulin light chain amyloidosis; ANCA, anti-neutrophil cytoplasmic antibody-associated glomerulonephritis; IgA, Immunoglobulin A nephropathy; MIgN, monoclonal immunoglobulin-related kidney disease; MN, membranous nephropathy; PE, preeclampsia.

Fig 6A. Congophilia of urine samples and HSA solution after centrifugation on concentrators with cut-offs of 30 and 100 kDa.

Samples were stained with Congo red, applied on nitrocellulose membrane, washed in ethanol and imaged with camera (Redmi Note 10, ISO 100) in lab-made box with two LED lamps. MW, molecular weight.

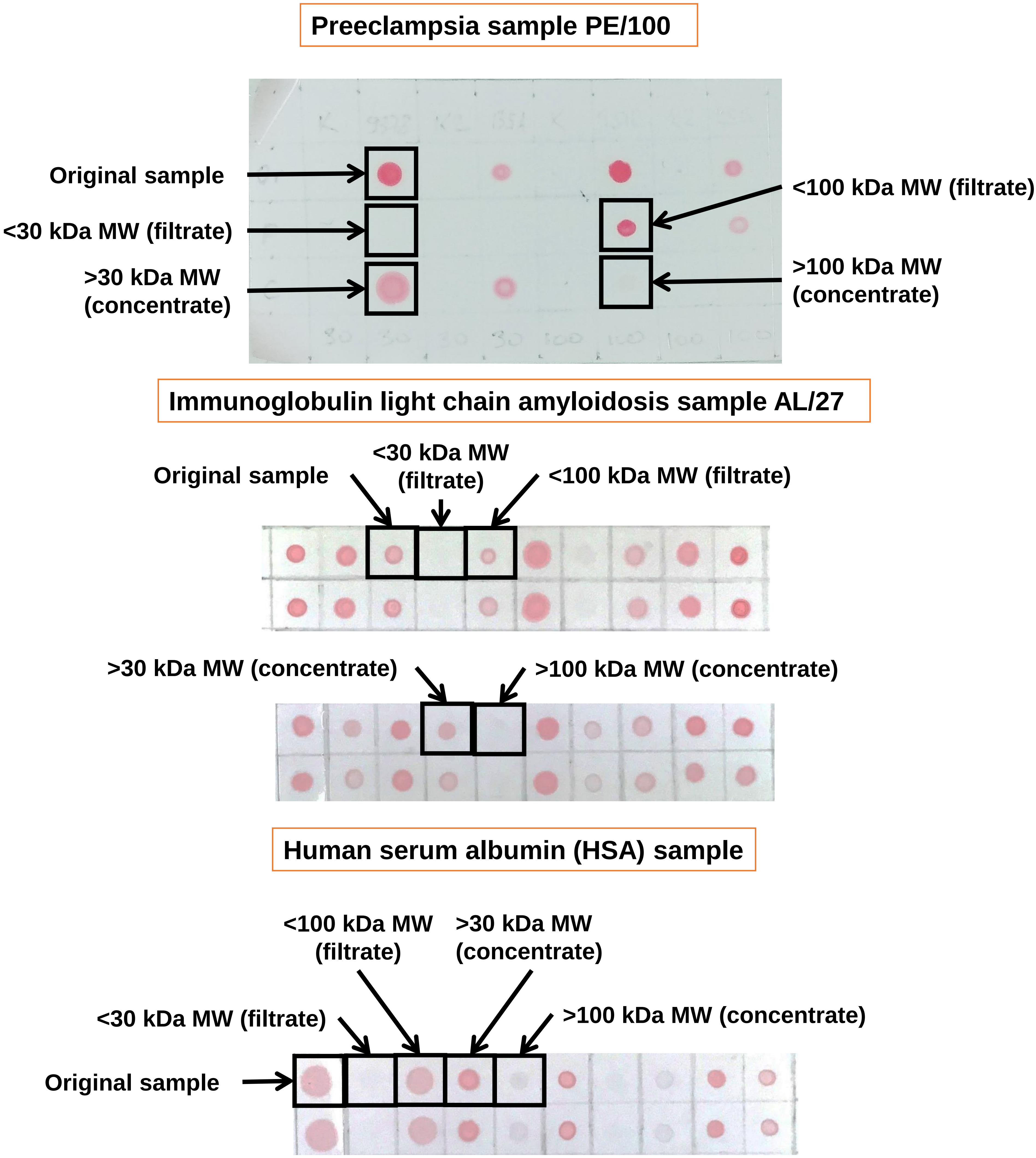

Fig 6B. 10% polyacrylamide gel electrophoresis of urine samples with 15 µg of protein is shown. Proteins in the gel are stained by Coomassie brilliant blue and imaged with ChemiDoc Imaging Systems (Bio-Rad, USA). Spectra Multicolor Broad Range Protein Ladder (26634, Thermo Scientific, USA) was used to assess molecular weight of proteins.

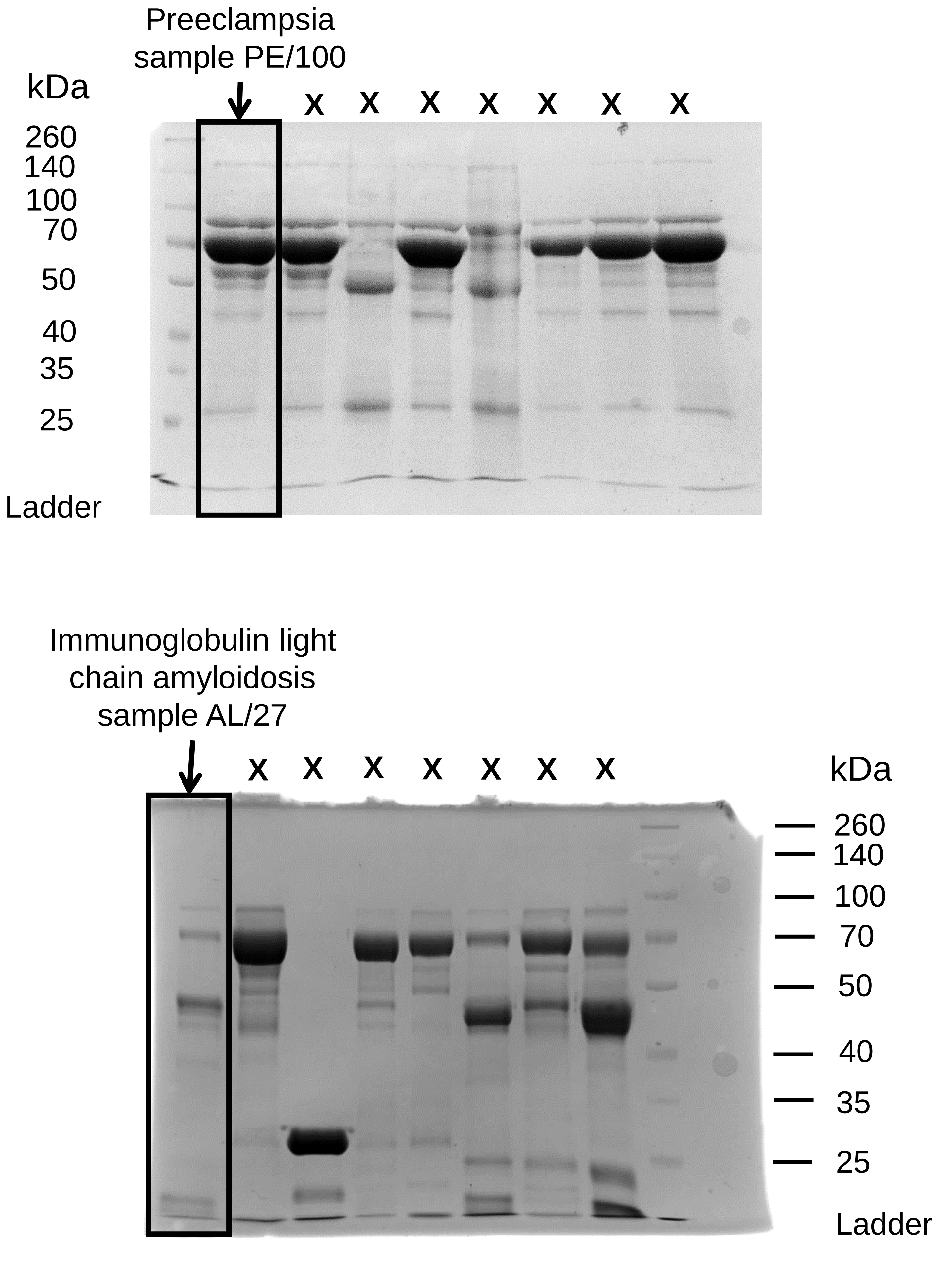

Fig 7C. 10 % polyacrylamide gel electrophoresis results for original and concentrated samples (1 µg of protein) obtained from four healthy pregnant women (NP/130-134). 2 µg of bovine serum albumin BSA was applied to localize the putative HSA in the analyzed samples. Proteins in the gel are stained by Coomassie brilliant blue and imaged with ChemiDoc Imaging Systems (Bio-Rad, USA). Spectra Multicolor Broad Range Protein Ladder (26634, Thermo Scientific, USA) was used to assess molecular weight of proteins.

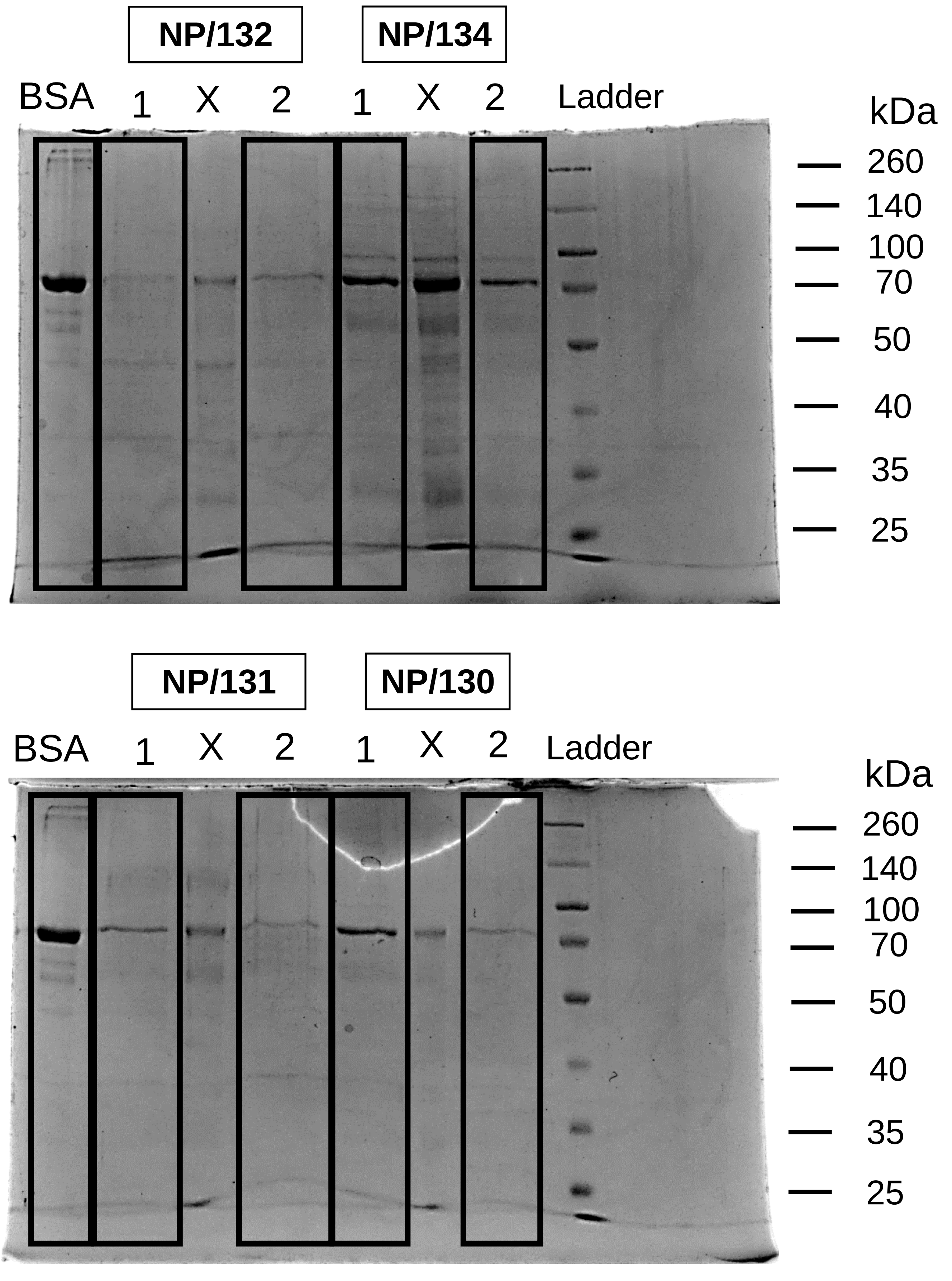

1 – concentrated samples
2 – non-concentrated samples
