# Supplementary material for: Diagnostics of preeclampsia based on Congo red binding to urinary components: Rationales and limitations
Source: PLoS One. 2024 Jan 19;19(1):e0297144. doi: 10.1371/journal.pone.0297144 (PMC10798433; doi:10.1371/journal.pone.0297144)

PLOS ONE

Diagnostics of preeclampsia based on Congo red binding to urinary components: rationales and limitations

--Manuscript Draft--

Manuscript Number:	PONE-D-23-29876
Article Type:	Research Article
Full Title:	Diagnostics of preeclampsia based on Congo red binding to urinary components: rationales and limitations
Short Title:	Congo red tests for diagnosis of preeclampsia
Corresponding Author:	Sergei Fedotov, Ph.D.<br>Pavlov Institute of Physiology of the Russian Academy of Sciences<br>Saint Petersburg, RUSSIAN FEDERATION
Keywords:	Congo red dot test; proteinuria; pregnancy complications; renal amyloidosis; nephropathies; amyloid-like aggregates
Abstract:	Preeclampsia is a disease in pregnancy that is one of the main causes of death in pregnant women. This disease occurs after the 20th week of pregnancy and is characterized by arterial hypertension, proteinuria, fetoplacental, and multiple organ dysfunctions. Despite the long history of studying preeclampsia, its etiology and pathogenesis remain poorly understood, and therapy is symptomatic. One of the factors in the development of the disease is presumably misfolded proteins that are prone to form amyloid aggregates. Diagnostic tests based on the binding of the amyloid-specific dye Congo red to urine components (CRD tests) show high efficiency in the diagnosis of preeclampsia. However, it was revealed that the CRD test gives a positive result in other diseases characterized by proteinuria, which is supposedly associated with the development of concomitant amyloidosis. This study aimed to assess the congophilia of urine samples from patients with different etiologies of proteinuria to determine the limitations of CRD tests. Patients with amyloidosis and non-amyloid nephropathy were analyzed. Congophilia values were shown to have a high correlation with urinary protein levels in all experimental groups. The mean values in patients diagnosed with amyloidosis did not differ from those in patients with proteinuria not associated with amyloid accumulation. Separation of urine fractions with different molecular weights indicates that the congophilia of urine samples is due primarily to binding to monomeric forms of the protein, including serum albumin. Our results confirm the presence of limitations in the use of the CRD tests in relation to pregnant women with renal disorders not associated with preeclampsia and amyloidosis and indicate the need for further study of the mechanisms of binding of Congo red with urine components.
Order of Authors:	Sergei A Fedotov, Ph.D.
	Maria S Khrabrova, Ph.D.
	Elena Vashukova
	Andrey Glotov
	Anastasia Anpilova
	Vladimir Dobronravov
	Maria Velizhanina
	Aleksandr A Rubel, Ph.D
Additional Information:	
Question	Response
**Financial Disclosure**<br><br>Enter a financial disclosure statement that	This study was supported by grant 22-25-00315 from Russian Science Foundation (MEV).<br>https://rscf.ru/project/22-25-00315/

Powered by Editorial Manager® and ProduXion Manager® from Aries Systems Corporation

describes the sources of funding for the work included in this submission. Review the submission guidelines for detailed requirements. View published research articles from *PLOS ONE* for specific examples.<br><br>This statement is required for submission and **will appear in the published article** if the submission is accepted. Please make sure it is accurate.<br><br>**Unfunded studies**<br>Enter: *The author(s) received no specific funding for this work.*<br><br>**Funded studies**<br>Enter a statement with the following details:<br>• Initials of the authors who received each award<br>• Grant numbers awarded to each author<br>• The full name of each funder<br>• URL of each funder website<br>• Did the sponsors or funders play any role in the study design, data collection and analysis, decision to publish, or preparation of the manuscript?<br>• **NO** - Include this sentence at the end of your statement: *The funders had no role in study design, data collection and analysis, decision to publish, or preparation of the manuscript.*<br>• **YES** - Specify the role(s) played.<br><br>* typeset	The funders had no role in study design, data collection and analysis, decision to publish, or preparation of the manuscript.
**Competing Interests**<br><br>Use the instructions below to enter a competing interest statement for this submission. On behalf of all authors, disclose any competing interests that could be perceived to bias this work—acknowledging all financial support and any other relevant financial or non-financial competing interests.<br><br>This statement is required for submission and **will appear in the published article** if the submission is accepted. Please make sure it is accurate and that any funding	The authors have declared that no competing interests exist.

sources listed in your Funding Information later in the submission form are also declared in your Financial Disclosure statement.<br><br>View published research articles from *PLOS ONE* for specific examples.<br><br>**NO authors have competing interests**<br><br>Enter: *The authors have declared that no competing interests exist.*<br><br>**Authors with competing interests**<br><br>Enter competing interest details beginning with this statement:<br><br>*I have read the journal's policy and the authors of this manuscript have the following competing interests: [insert competing interests here]*<br><br>* typeset	
**Ethics Statement**<br><br>Enter an ethics statement for this submission. This statement is required if the study involved:<br><br>• Human participants<br>• Human specimens or tissue<br>• Vertebrate animals or cephalopods<br>• Vertebrate embryos or tissues<br>• Field research<br><br>Write "N/A" if the submission does not require an ethics statement.<br><br>General guidance is provided below. Consult the submission guidelines for detailed instructions. **Make sure that all information entered here is included in the Methods section of the manuscript.**	All participants in the study signed the written informed consent. The study was approved either by the Ethics Committees of Pavlov University (St. Petersburg, Russia) (No.: 21-250; date of approval 28 June 2021) or the Research Institute of Obstetrics (St. Petersburg, Russia) (No.: 97; date of approval 27 June 2019).

**Format for specific study types**<br><br>**Human Subject Research (involving human participants and/or tissue)**<br>• Give the name of the institutional review board or ethics committee that approved the study<br>• Include the approval number and/or a statement indicating approval of this research<br>• Indicate the form of consent obtained (written/oral) or the reason that consent was not obtained (e.g. the data were analyzed anonymously)<br><br>**Animal Research (involving vertebrate animals, embryos or tissues)**<br>• Provide the name of the Institutional Animal Care and Use Committee (IACUC) or other relevant ethics board that reviewed the study protocol, and indicate whether they approved this research or granted a formal waiver of ethical approval<br>• Include an approval number if one was obtained<br>• If the study involved *non-human primates*, add *additional details* about animal welfare and steps taken to ameliorate suffering<br>• If anesthesia, euthanasia, or any kind of animal sacrifice is part of the study, include briefly which substances and/or methods were applied<br><br>**Field Research**<br><br>Include the following details if this study involves the collection of plant, animal, or other materials from a natural setting:<br>• Field permit number<br>• Name of the institution or relevant body that granted permission	
**Data Availability**<br><br>Authors are required to make all data underlying the findings described fully available, without restriction, and from the time of publication. PLOS allows rare exceptions to address legal and ethical concerns. See the PLOS Data Policy and FAQ for detailed information.	Yes - all data are fully available without restriction

Powered by Editorial Manager® and ProduXion Manager® from Aries Systems Corporation

A Data Availability Statement describing where the data can be found is required at submission. Your answers to this question constitute the Data Availability Statement and **will be published in the article**, if accepted.<br><br>**Important:** Stating 'data available on request from the author' is not sufficient. If your data are only available upon request, select 'No' for the first question and explain your exceptional situation in the text box.<br><br>Do the authors confirm that all data underlying the findings described in their manuscript are fully available without restriction?	
**Describe where the data may be found in full sentences. If you are copying our sample text, replace any instances of XXX with the appropriate details.**<br><br>• If the data are **held or will be held in a public repository**, include URLs, accession numbers or DOIs. If this information will only be available after acceptance, indicate this by ticking the box below. For example: *All XXX files are available from the XXX database (accession number(s) XXX, XXX.*).<br>• If the data are all contained **within the manuscript and/or Supporting Information files**, enter the following: *All relevant data are within the manuscript and its Supporting Information files.*<br>• If neither of these applies but you are able to provide **details of access elsewhere**, with or without limitations, please do so. For example:<br><br>*Data cannot be shared publicly because of [XXX]. Data are available from the XXX Institutional Data Access / Ethics Committee (contact via XXX) for researchers who meet the criteria for access to confidential data.*<br><br>*The data underlying the results presented in the study are available from (include the name of the third party*	All relevant data are within the manuscript and its Supporting Information files.

and contact information or URL).<br>• This text is appropriate if the data are owned by a third party and authors do not have permission to share the data.<br><br>* typeset	
Additional data availability information:	

Manuscript Click here to access/download;Manuscript;Manuscript.docx

Diagnostics of preeclampsia based on Congo red binding to urinary components: rationales and limitations

Short title

Congo red tests for diagnosis of preeclampsia

Sergei A. Fedotov [1, 2, 3]*, Maria S. Khrabrova [1, 4], Elena S. Vashukova [5], Andrey S. Glotov [5], Anastasia O. Anpilova [1, 6], Vladimir A. Dobronravov [6], Maria E. Velizhanina [1], Aleksandr A. Rubel [1, 7]*

1 Laboratory of Amyloid Biology, Saint Petersburg University, St. Petersburg, Russia

2 Laboratory of Toxinology and Molecular Systematics, L.A. Orbeli Institute of Physiology, National Academy of Sciences of the Republic of Armenia, Yerevan, Armenia

3 Laboratory of Comparative Behavioral Genetics, Pavlov Institute of Physiology, Russian Academy of Sciences, St. Petersburg, Russia

4 Department of Propaedeutics of Internal disease, Pavlov University, St. Petersburg, Russia

5 Department of Genomic Medicine, D.O. Ott Research Institute of Obstetrics, Gynecology and Reproductology, St. Petersburg, Russia

6 Research Institute of Nephrology, Pavlov University, St. Petersburg, Russia

7 Department of Genetics and Biotechnology, St. Petersburg State University, St. Petersburg, Russia

*Corresponding authors

E-mail: serg900@yandex.ru (SAF)

E-mail: a.rubel@spbu.ru (AAR)

Abstract

Preeclampsia is a disease in pregnancy that is one of the main causes of death in pregnant women. This disease occurs after the 20th week of pregnancy and is characterized by arterial hypertension, proteinuria, fetoplacental, and multiple organ dysfunctions. Despite the long history of studying preeclampsia, its etiology and pathogenesis remain poorly understood, and therapy is symptomatic. One of the factors in the development of the disease is presumably misfolded proteins that are prone to form amyloid aggregates. Diagnostic tests based on the binding of the amyloid-specific dye Congo red to urine components (CRD tests) show high efficiency in the diagnosis of preeclampsia. However, it was revealed that the CRD test gives a positive result in other diseases characterized by proteinuria, which is supposedly associated with the development of concomitant amyloidosis. This study aimed to assess the congophilia of urine samples from patients with different etiologies of proteinuria to determine the limitations of CRD tests. Patients with amyloidosis and non-amyloid nephropathy were analyzed. Congophilia values were shown to have a high correlation with urinary protein levels in all experimental groups. The mean values in patients diagnosed with amyloidosis did not differ from those in patients with proteinuria not associated with amyloid accumulation. Separation of urine fractions with different molecular weights indicates that the congophilia of urine samples is due primarily to binding to monomeric forms of the protein, including serum albumin. Our results confirm the presence of limitations in the use of the CRD tests in relation to pregnant women with renal disorders not associated with preeclampsia and amyloidosis and indicate the need for further study of the mechanisms of binding of Congo red with urine components.

Introduction

Preeclampsia is a severe complication of pregnancy that is characterized by elevated blood pressure, proteinuria, seizures, failure of multiple organs, and even death [1–7]. An accurate and early diagnosis of this condition is an unmet clinical need [8,9]. In 2014, Buhimschi et al. demonstrated that urine proteins in patients with preeclampsia exhibited properties similar to amyloid [10]. For diagnostic

purposes, they developed the Congo red dot (CRD) test, which assessed the retention of the amyloid-specific Congo red dye by urine proteins on a membrane (Congo red retention, CRR). Samples from preeclampsia patients retained the dye, while those from non-preeclampsia patients lost it when washed off (Fig. 1A). For use in clinical practice modified versions of the CRD test have been introduced known as CRD paper tests [11,12] where positive test results are determined by spot area (Fig. 1B). When tested in pregnant women, this modified test provided a sensitivity of 80.2% and specificity of 89.2%, making it more effective than well-known disease markers such as fms-like tyrosine kinase 1 and placental growth factor [11].

Fig 1. The representation of the CRD tests' results. The CRD membrane (A) and CRD paper (B) tests are shown. The representative images displayed are from five pregnant women with preeclampsia (1–5) and five control pregnant women (6–10).

Recently, McCarthy et al. assessed the effectiveness of CRD tests in pregnant and non-pregnant women with chronic kidney disease (CKD) with unspecified morphology, hypertension, and lupus nephritis [13]. In this study, the CRD test was positive in all patients with proteinuria, regardless of the diagnosis. Whether the renal amyloidoses and other proteinuric nephropathies yield CRD test positivity remains unknown. Another poorly investigated aspect [10] is whether urinary congophilia in patients with preeclampsia originates from urinary proteins themselves or amyloid-like aggregates. To address these gaps and identify potential limitations of the CRD test, we investigated congophilia and protein components mediating Congo red positivity in urine samples from patients with proteinuria of different etiology, including preeclampsia and histologically confirmed amyloid and non-amyloid nephropathies.

Materials and methods

Study design, patient selection, and data collection

Two study groups comprised patients with amyloid nephropathies (AN) and non-amyloid nephropathies (NA), confirmed by detailed clinical and histological evaluation. We also enrolled women with

preeclampsia (PE) as the positive controls. Women with normal pregnancy (NP) and patients with various non-proteinuric kidney diseases (Control) served as negative controls. Preeclampsia was diagnosed according to the clinical guidelines [7]. Inclusion criteria for Control and NP were 24-h albuminuria <30 mg and estimated glomerular filtration rate (eGFR) >60 ml/min/1,73m^2. At the time of enrollment, pregnancy was excluded in all women from the AN, NA, and Control groups. Serum creatinine was used to evaluate kidney function [14], while the eGFR was additionally calculated using the CKD-EPI 2009 formula [15] for the AN, NA, and Control groups. To assess proteinuria, the Quick Start Bradford Protein Assay kit (Bio-Rad, USA) was used.

All participants signed the informed consent as a part of Regular Medical documentation. The study was approved either by the Ethics Committees of Pavlov University (St. Petersburg, Russia) (No.: 21-250; date of approval 28 June 2021) or the Research Institute of Obstetrics (St. Petersburg, Russia) (No.: 97; date of approval 27 June 2019). Urine samples for this study were collected from 14.09.2019 to 30.12.2021 for PE and NP groups and from 12.09.2021 to 28.12.2021 for AN, NA, and Control groups.

Congophilia assays

The urine samples were stored at -80 °C, thawed on ice, and centrifuged at 4000 x*g*, 4 °C for 5 min before the experiments. In congophilia tests, 80 µl of each sample was mixed with 8 µl of a 0.2 % aqueous solution of Congo red dye, incubated for 10 min and applied on a nitrocellulose membrane (Amersham Protran 0.45 NC, GE Healthcare, USA) in two aliquots of 2 µl (membrane test) and FN 3 chromatography paper (thickness: 0.19 mm; square weight: 90 g/m^2) in two aliquots of 40 µl (paper test). The membrane was dried for 10 minutes, wetted with water, imaged using a camera in a lab-made box, then incubated for 1 min in 50 % and 70 % ethanol, and washed for 1 h in 90% ethanol with shaking. After rinsing successively in 70 % ethanol, 50 % ethanol, and water, the membrane was imaged again. The integrated intensity of each spot was determined using ImageJ (version 1.51j8) and the CRR was calculated as the ratio of the average intensities after washing and before washing. In the paper test, the stained samples were scanned 15 min after application on the paper, and the area of the spots was calculated in ImageJ as Congo red area (CRA, px).

For congophilia tests and polyacrylamide gel electrophoresis (PAGE), the following proteins were used: human serum albumin (HSA) (A3782, Sigma-Aldrich, USA), bovine serum albumin (BSA) (23209, Thermo Fisher Scientific, USA), and bovine gamma globulin (BGG) (500-0208, Bio-Rad, USA).

Separation of urine fractions

The separation of high molecular weight (MW) aggregates was carried out using an Optima MAX-XP ultracentrifuge (Beckman Coulter, USA) at 300,000 x*g* and 4 °C for 2 h. To isolate protein fractions within specific MW ranges, 200 µl of urine samples were applied onto Amicon Ultra-4 Centrifugal Filter Devices (Merck Millipore, Germany) with a cut-off of 30 kDa (UFC8030) and 100 kDa (UFC8100), followed by centrifugation at 7,000 x*g* for 10 min. Subsequently, 190 µl of filtrates and 10 µl of concentrates were collected (concentration factor 20). Concentrates were diluted by phosphate buffer, pH 7.4 (Merck, S-P4417) to 200 µl, concentrated once more (washing), and diluted to the volume of the original sample.

Urine protein concentrating

Control urine samples (20-80 ml) of NP were lyophilized in a freeze dryer (Labconco Corporation, USA), dissolved in water to a volume of 1.5-2 ml, and centrifuged for 10 min at 4,000 x*g*. The supernatant was analyzed by 10% PAGE followed by Coomassie brilliant blue staining. Spectra Multicolor Broad Range Protein Ladder (26634, Thermo Scientific, USA) was used to assess MW of proteins separated in PAGE.

Statistics

Data are presented as median with interquartile range or mean ± standard deviation for continuous variables and frequencies with % for categorical variables. Parameters among the groups were compared by analysis of variance. For continuous and categorical variables comparison, the Mann-Whitney U-test and the chi-square test were applied, respectively. The mean CRRs, CRAs, and proteinuria were compared between groups by a two-sided randomization test in the Drosophila Courtship Lite v. 1.3 [16,17]. The 95 % confidence intervals (C. I.) for means were calculated by bootstrapping (10,000 iterations) [18]. The 95 % C. I. and *p*-values for the Spearman rank-order

correlation coefficient were assessed by *t*-test. To determine the linear approximation (coefficient of determination, R^2) Microsoft Excel 2016 was used. Statistical significance was assumed at $p < 0.01$.

Results

Patient description

AN group presented with 5 cases of serum amyloid A amyloidosis and 22 cases of immunoglobulin light chain (AL)-amyloidosis. NA group included patients with focal segmental glomerulosclerosis (n = 5), non-amyloid type of monoclonal immunoglobulin-related kidney disease (n = 5), membranous nephropathy (n = 4), diabetic nephropathy (n = 4), immunoglobulin A nephropathy (n = 4), anti-neutrophil cytoplasmic antibodies associated glomerulonephritis (n = 2), lupus nephritis (n = 1), C3-glomerulopathy (n = 1), and idiopathic membranoproliferative glomerulonephritis (n = 1). The control group comprised patients with diabetes mellitus (n = 13), cardiovascular disease (n = 12), systemic autoimmune disorder (n = 2), aplastic anemia (n = 2), Cushing disease (n = 1), human immunodeficiency virus (n = 1), multiple myeloma (n = 1), and four healthy persons. Demographic and clinical findings of the studied groups are presented in Table 1.

Table 1. Demographic and clinical data in studied groups

**Parameters[a]**	**AN (n = 27)**	**NA (n = 27)**	**Control (n = 36)**	**PE (n = 13)**	**NP (n = 31)**
**Woman/man, n/n**	18/9	15/12	24/12	13/0	31/0
**Age, years**	59±11	49±15	48±17	32±4	32±4
**BMI, kg/m^2**	24.2 (20.4; 25.9)	25.4 (22.1; 30.3)	27.2 (23.0; 31.0)	30 (26; 36)	25 (24; 28)
**Serum creatinine, mg/dl[b]**	1.13 (0.9; 2.77)	1.55 (0.9; 2.23)	0.81 (0.69; 0.94)	0.76 (0.7; 0.8)	0.73 (0.68; 0.81)
**eGFR CKD-EPI, ml/min/1,73m^2 [b]**	54 (23; 86)	43 (27; 95)	85 (76; 102)	n/a	n/a
**24-h proteinuria, g/24-h**	8 (5.6;12.4)	6.6 (3.6; 13.1)	n/a	5 (2.5; 5.5)	n/a

**24-h albuminuria, mg/24-h**	n/a	n/a	8 (6;14)	n/a	10 (6; 15)

AN, amyloid nephropathies; BMI, body mass index; Control, patients without proteinuria; eGFR, estimated glomerular filtration rate; NA, non-amyloid nephropathies; NP, normal pregnancy; PE, preeclampsia.

[a] No parameters differed significantly between AN and NA when compared in pairs, as well as between PE and NP.

[b] In the ANOVA analysis, the Control group significantly differed from AN and NA in terms of eGFR and serum creatinine (both with $p < 0.001$).

Congophilia of urine samples did not differ in groups of amyloid and non-amyloid nephropathies

The mean CRAs in the AN and NA groups were 2.5 and 2.2 times higher, respectively, compared with the Control group (Fig. 2A). Similarly, the mean CRA was two-fold higher in PE compared with the NP group. The mean CRAs did not differ between AN and NA groups. This trend was consistent when comparing mean CRR in the membrane test (Fig. 2B) and protein concentrations between groups (Fig. 2C).

Fig 2. The results of the CRD tests and proteinuria levels in studied groups. The values of congophilia observed in the paper (A) and membrane (B) tests, as well as the levels of proteinuria (C), are shown. The box plots present the 25th and 75th percentiles (box), the maximum and minimum values, the median (line in the box), and the mean (cross). AN, amyloid nephropathies; Control, patients without proteinuria; CRA, Congo red area; CRR, Congo red retention; NA, non-amyloid nephropathies; NP, normal pregnancy; PE, preeclampsia.

The urine protein excretion and CRR values strongly correlated in AN, NA, and PE groups (Fig. 3).

Fig 3. The quantitative relationship between urine congophilia and proteinuria in three groups with nephropathies. The Spearman rank-order correlation coefficients (Spearman's correlation), 95 % confidence intervals (C. I.), *p* values (*t*-test), and scatter charts with linear trend lines are shown for each group. AN, amyloid nephropathies; CRR, Congo red retention; NA, non-amyloid nephropathies; PE, preeclampsia.

To examine Congo red binding to the main urine proteins, we conducted the CRD membrane tests on a series of dilutions of HSA and BGG in phosphate buffer, pH 7.4, and revealed a positive correlation

between CRR and the concentration of these proteins (Fig. 4A and 4B). The average ratio of CRRs to protein levels in HSA samples and the PE group did not differ (Fig. 4C).

Fig 4. The quantitative relationship between congophilia and concentrations of BGG and HSA in comparison with preeclampsia samples. The reliability of the linear approximation (coefficient of determination, R^2) of CRR dependence on concentrations of BSA (A) and HSA (B) is shown. Section B also shows urinary congophilia in the PE group (grey circles). (C) The ratios of the CRRs to the protein concentrations in HSA solutions and PE samples are shown. CRR, Congo red retention; HSA, human serum albumin; PE, preeclampsia.

Urinary congophilia was determined by binding to proteoforms of the 30-100 kDa range

The contribution of large urine protein aggregates to CRR value and the MW of the urine proteins binding to Congo red were estimated. After isolating the aggregates from urine samples by ultracentrifugation, the CRR values of the supernatants still were high. The mean CRRs did not differ between supernatants after centrifugation at 300,000 x*g* and 4,000 x*g* (p = 0.432, Fig. 5).

Fig. 5. Congophilia of urine samples from patients of different groups before and after ultracentrifugation. On the left, sample preparation conditions, mean CRRs (n = 11), and 95 % C. I. are indicated. AA, serum amyloid A amyloidosis; AL, Immunoglobulin light chain amyloidosis; ANCA, anti-neutrophil cytoplasmic antibody-associated glomerulonephritis; CF, centrifugation; CRR, Congo red retention; IgA, Immunoglobulin A nephropathy; MM, multiple myeloma; MN, membranous nephropathy; PE, preeclampsia.

Separation of protein fractions in HSA solution and urine samples using the centrifugal concentrators revealed congophilic components within the 30-100 kDa range (Fig. 6A). According to PAGE analysis, the main two protein bands in the urine samples were around 45-50 and 70 kDa (Fig. 6B).

Fig 6. Congophilia of urine samples and HSA solution after centrifugation on concentrators with cut-offs of 30 and 100 kDa. (A) Samples before centrifugation (original samples) and after centrifugation (concentrates and filtrates) are analyzed using a membrane test. At the left, urine protein fractions are listed. (B) 10 % polyacrylamide gel electrophoresis of urine samples with 15 µg of protein is shown. Proteins in the gel are stained by Coomassie

brilliant blue. AL, Immunoglobulin light chain amyloidosis; HSA, human serum albumin; MW, molecular weight; PE, preeclampsia.

Concentrated urine was less congophilic than HSA solution

The effectiveness of the CRD tests was previously assessed by utilizing concentrated urine samples from healthy pregnant women as control subjects [10]. We compared the CRR values between concentrated urine samples and an HSA solution with the same protein concentration given that the ratio of congophilia to protein is equal in non-concentrated samples and the HSA solutions (Fig. 3). Four control urine samples of pregnant women with a protein level less than 0.3 mg/ml were concentrated by lyophilization, dissolved in water, and centrifuged at 4,000 x*g*. The volume of the supernatant was approximately equal to that of the precipitate, and its protein concentration was approximately 2.5 times lower than the concentration in the samples before lyophilization multiplied by the concentration factor (Fig. 7A). Thus, the majority of the protein after dissolution remained in the sediment. The membrane test showed a lower CRR in each concentrated sample (supernatant) compared to an equal concentration of HSA (Fig. 7B). PAGE analysis of the original and concentrated samples revealed no apparent qualitative changes in the protein composition. In nearly all cases, a distinct band, presumably attributed to HSA, was observed (Fig. 7C).

Fig 7. Congophilia and protein composition in concentrated urine samples from women with uncomplicated pregnancy. (A) From left to right: initial protein concentrations and protein amounts in the four control urine samples (Ctrl1-4); procedure scheme; protein concentrations and total amounts of protein (weights and percentages of the initial amount) in the supernatant obtained by concentrating. (B) Mean CRRs of original samples, concentrated samples, and HSA solutions at concentrations equal to concentrated samples are shown. (C) 10 % polyacrylamide gel electrophoresis results for original and concentrated samples (1 µg of protein) are shown. BSA (2 µg) was applied to localize the putative HSA in the analyzed samples. Proteins in the gel are stained by Coomassie brilliant blue. BSA, bovine serum albumin; CF, centrifugation; CRR, Congo red retention; Ctrl, control sample from a woman with uncomplicated pregnancy; RT, room temperature.

Discussion

Our study was the first to evaluate urine congophilia using the CRD test in morphologically verified proteinuric nephropathies, including non-amyloid diseases and amyloidosis. The latter is assumed to be more prone to urine congophilia [11]. The study's findings demonstrated that the CRD test yielded positive results in all proteinuric patients, including male patients and those with non-amyloid kidney diseases. Urinary congophilia was strongly correlated with the urine protein concentration, regardless of the etiology of proteinuria and sex (Fig. 2 and 3). The positivity of the CRD test in cases with proteinuria limits its clinical utility for diagnosing preeclampsia. Thus, pregnant women with pre-existing renal disease unrelated to pregnancy can yield positive results in the CRD tests. Conversely, when proteinuria is absent in cases of preeclampsia, the CRD test may also have reduced effectiveness, as evidenced by a recent meta-analysis [19].

Similar to our findings, the Buhimschi group demonstrated a correlation between proteinuria and urinary congophilia and suggested a formation of amyloid-like protein aggregates as substrates for Congo red binding [10]. In our experiments, urinary congophilia was associated with urine proteins with a MW less than 100 kDa (Fig. 6), and the CRD test was still positive after removing possible aggregates of urine proteins (Fig. 5). These findings make unlikely the CRD test positivity to be attributed to large protein aggregates. As serum albumin and immunoglobulins demonstrate congophilia (Fig. 4) and are typical in urines in various nephropathies [20,21], these proteins might be responsible for the urinary congophilia in any proteinuric conditions. The binding of Congo red to HSA monomers [22] and the presence of urinary congophilia only in the 30-100 kDa fraction (Fig. 6A) confirm our assumption. However, we cannot entirely rule out the presence of low-molecular protein aggregation either under specific urine microenvironments or induced by conditions of the CRD test conducting.

To demonstrate the efficacy of the CRD test previous studies compared urinary congophilia in samples from preeclampsia patients with samples from normally pregnant women which were preliminary concentrated to achieve the same concentration of proteins in both groups [10,13]. We showed that the lower congophilia observed in the healthy pregnant might be attributed to a distinct qualitative protein composition before and after concentrating since the proteins could potentially precipitate out (Fig. 7).

Our results shed light on the possible reasons for the inaccurate estimation of the CRD test effectiveness.

Conclusion

Tests for congophilia in diagnosing preeclampsia are simple, cheap, and fast for primary screening of pregnant women [23,24]. At the same time, the effectiveness of these methods can vary greatly in hospitals in different countries [23]. Our study showed that urinary congophilia is largely determined by protein concentration and is not associated with the presence of large protein aggregates in the sample. Positive test results are obtained for patients with renal disorders of various etiologies, some of which are not associated with the detection of amyloid deposits in the body. Further research focusing on molecular mechanisms of Congo red binding to specific urine proteins could propose the possible clinical applications of the congophilia phenomenon in preeclampsia and other nephropathies.

Acknowledgments

The authors wish to express their gratitude to Dr. Yury O. Chernoff from the School of Biological Sciences at Georgia Institute of Technology, USA, for his consultation, criticism, and valuable comments. The authors acknowledge the support from St. Petersburg State University (project 94031363) and the Center for Molecular and Cell Technologies (Research Park, St. Petersburg State University). All individuals and organizations consent to the acknowledgment and report no funding or conflict of interest except already mentioned above.

References

1. Redman CW, Sargent IL. Latest Advances in Understanding Preeclampsia. Science. 2005 Jun 10;308(5728):1592–4.

2. Hypertension in pregnancy. Report of the American College of Obstetricians and Gynecologists' Task Force on Hypertension in Pregnancy. Obstet Gynecol. 2013 Nov;122(5):1122–31.

3. Brown MA, Magee LA, Kenny LC, Karumanchi SA, McCarthy FP, Saito S, et al. Hypertensive Disorders of Pregnancy.

4. Duley L. The Global Impact of Pre-eclampsia and Eclampsia. Seminars in Perinatology. 2009 Jun;33(3):130–7.

5. Sircar M, Thadhani R, Karumanchi SA. Pathogenesis of preeclampsia: Current Opinion in Nephrology and Hypertension. 2015 Mar;24(2):131–8.

6. Malik A, Jee B, Gupta SK. Preeclampsia: Disease biology and burden, its management strategies with reference to India. Pregnancy Hypertension. 2019 Jan;15:23–31.

7. Gestational Hypertension and Preeclampsia: ACOG Practice Bulletin, Number 222. Obstetrics & Gynecology. 2020 Jun;135(6):e237.

8. Backes CH, Markham K, Moorehead P, Cordero L, Nankervis CA, Giannone PJ. Maternal Preeclampsia and Neonatal Outcomes. Journal of Pregnancy. 2011;2011:1–7.

9. Kosińska-Kaczyńska K, Wielgoś M. How to identify pregnant women at risk of pre-eclampsia? — a review of the current literature. Ginekol Pol. 2018 Jun 29;89(6):335–8.

10. Buhimschi IA, Nayeri UA, Zhao G, Shook LL, Pensalfini A, Funai EF, et al. Protein misfolding, congophilia, oligomerization, and defective amyloid processing in preeclampsia. Sci Transl Med [Internet]. 2014 Jul 16 [cited 2022 Aug 29];6(245). Available from: https://www.science.org/doi/10.1126/scitranslmed.3008808

11. Rood KM. Congo Red Dot Paper Test for Antenatal Triage and Rapid Identification of Preeclampsia. 2019;10.

12. Li XM, Liu XM, Xu J, Du J, Cuckle H. Late pregnancy screening for preeclampsia with a urinary point-of-care test for misfolded proteins. Spradley FT, editor. PLoS ONE. 2020 May 20;15(5):e0233214.

13. McCarthy FP, Adetoba A, Gill C, Bramham K, Bertolaccini M, Burton GJ, et al. Urinary congophilia in women with hypertensive disorders of pregnancy and preexisting proteinuria or hypertension. American Journal of Obstetrics and Gynecology. 2016 Oct;215(4):464.e1-464.e7.

14. Wiles K, Chappell L, Clark K, Elman L, Hall M, Lightstone L, et al. Clinical practice guideline on pregnancy and renal disease. BMC Nephrol. 2019 Dec;20(1):401.

15. Levin A, Stevens PE, Bilous RW, Coresh J, Francisco ALMD, Jong PED, et al. Kidney disease: Improving global outcomes (KDIGO) CKD work group. KDIGO 2012 clinical practice guideline for the evaluation and management of chronic kidney disease. Kidney International Supplements. 2013 Jan 1;3(1):1–150.

16. Nuzzo RL. Randomization Test: An Alternative Analysis for the Difference of Two Means. PM&R. 2017 Mar;9(3):306–10.

17. Kamyshev NG, Iliadi KG, Bragina JV. *Drosophila* Conditioned Courtship: Two Ways of Testing Memory. Learn Mem. 1999 Jan 1;6(1):1–20.

18. Mokhtar SF, Md Yusof Z, Sapiri H. Confidence Intervals by Bootstrapping Approach: A Significance Review. Mal J Fund Appl Sci. 2023 Feb 25;19(1):30–42.

19. Khaliq ZM, Gouwens NW, Raman IM. The contribution of resurgent sodium current to high-frequency firing in Purkinje neurons: an experimental and modeling study. The Journal of neuroscience. 2003;23(12):4899–912.

20. D’Amico G, Bazzi C. Pathophysiology of proteinuria. Kidney International. 2003 Mar;63(3):809–25.

21. Patel DN, Kalia K. Characterization of low molecular weight urinary proteins at varying time intervals in type 2 diabetes mellitus and diabetic nephropathy patients. Diabetol Metab Syndr. 2019 Dec;11(1):39.

22. Zhang YZ, Xiang X, Mei P, Dai J, Zhang LL, Liu Y. Spectroscopic studies on the interaction of Congo Red with bovine serum albumin. Spectrochimica Acta Part A: Molecular and Biomolecular Spectroscopy. 2009 May;72(4):907–14.

23. Bracken H, Buhimschi IA, Rahman A, Smith PRS, Pervin J, Rouf S, et al. Congo red test for identification of preeclampsia: Results of a prospective diagnostic case-control study in Bangladesh and Mexico. EClinicalMedicine. 2021 Jan;31:100678.

24. Sailakshmi MPA, Prabhu MR, Prabhakara S, Anbazhagan K, Rupakala BM. Congo red dot test in the early prediction and diagnosis of pre-eclampsia in a tertiary health care centre in India. Pregnancy Hypertension. 2021 Aug;25:225–9.

Figure 1

Click here to access/download;Figure;Fig1.tif.tif

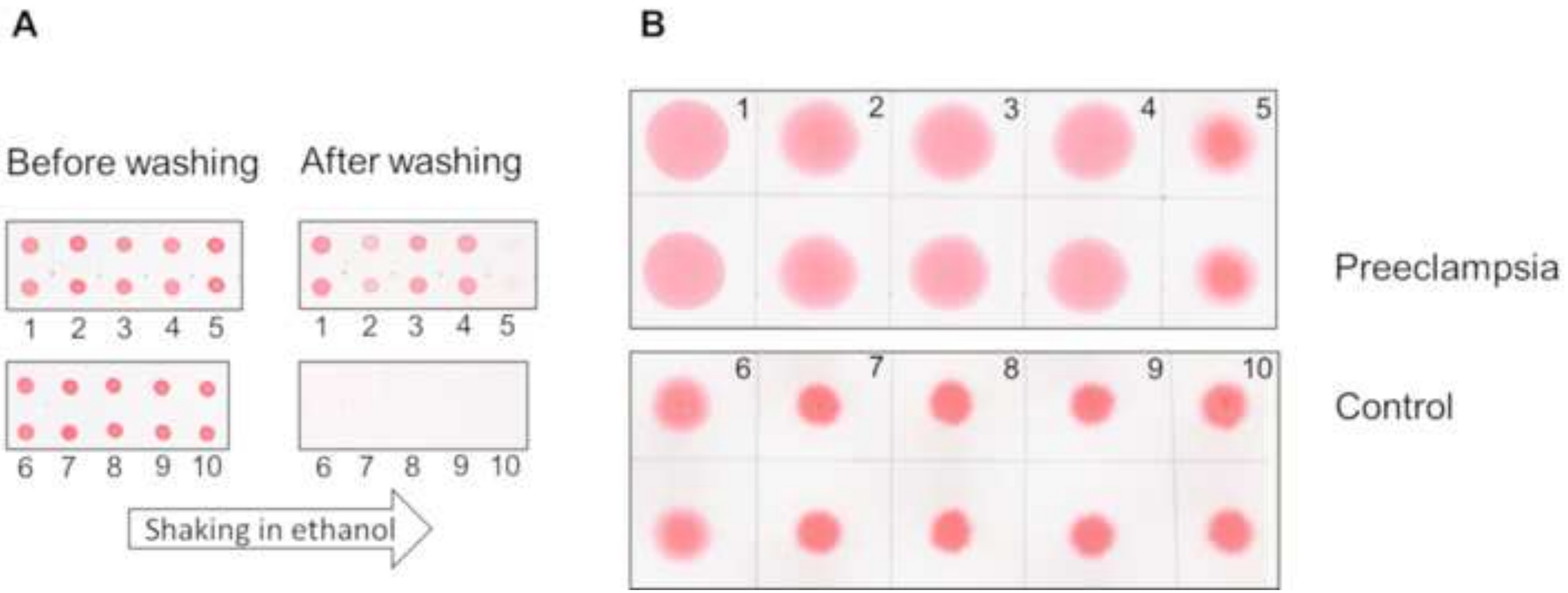

Figure 2

Click here to access/download;Figure;Fig2.tif.tif

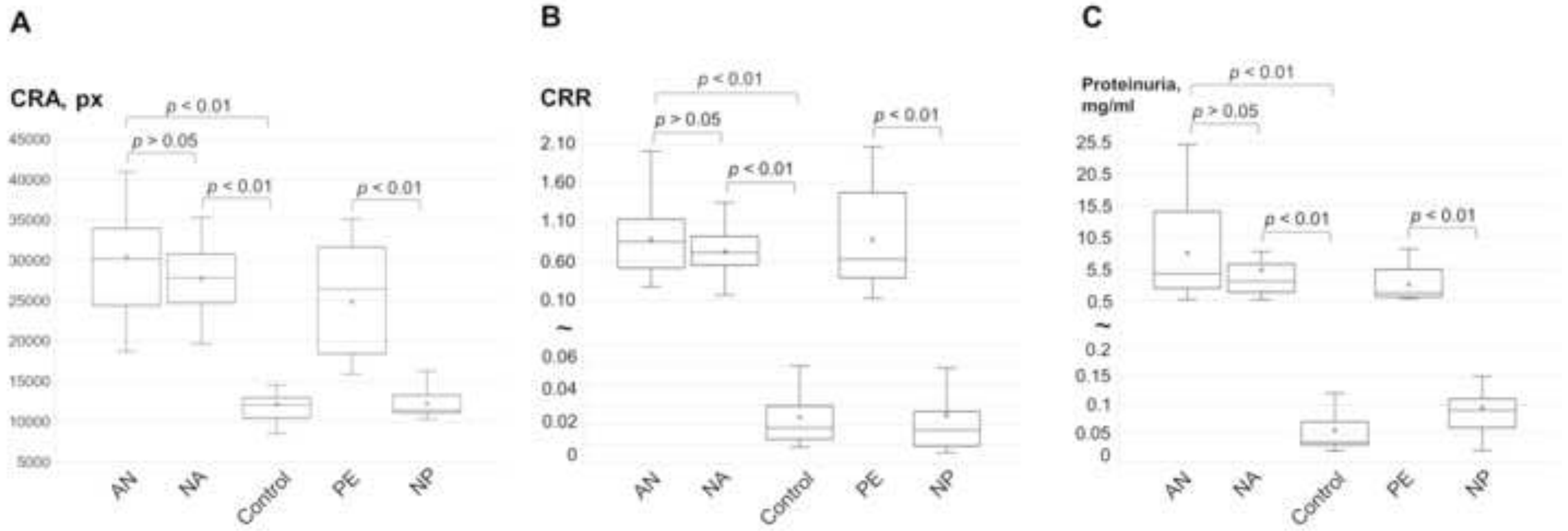

Figure 3

Click here to access/download;Figure;Fig3.tif.tif

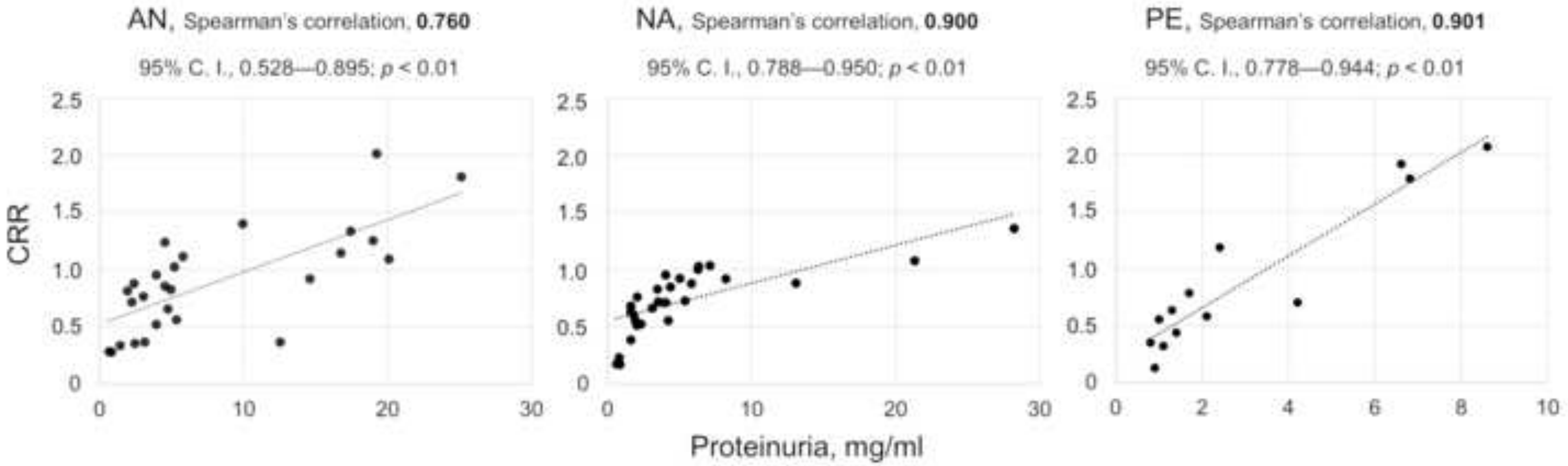

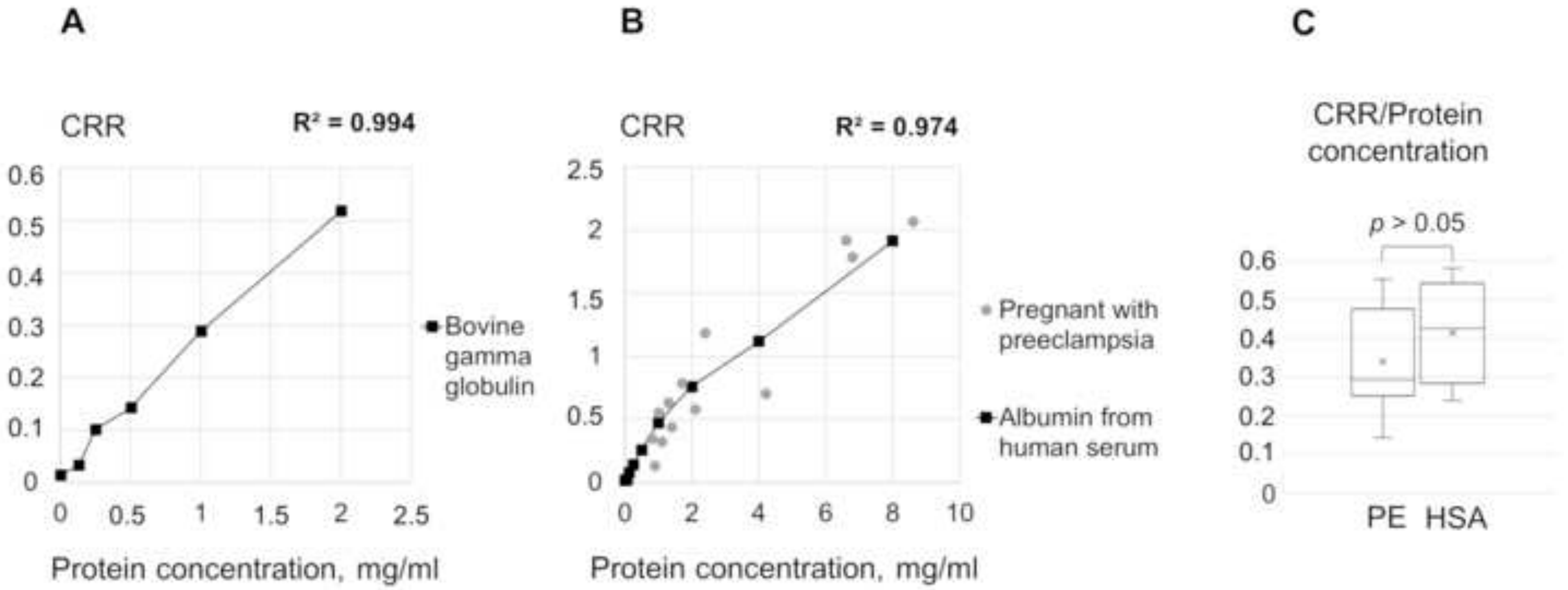
A
CRR
R² = 0.994
0.6
0.5
0.4
0.3
0.2
0.1
0
0
0.5
1
1.5
2
2.5
Bovine gamma globulin
Protein concentration, mg/ml
B
CRR
R² = 0.974
2.5
2
1.5
1
0.5
0
0
2
4
6
8
10
Pregnant with preeclampsia
Albumin from human serum
Protein concentration, mg/ml
C
CRR/Protein concentration
p > 0.05
0.6
0.5
0.4
0.3
0.2
0.1
0
PE
HSA

Figure 5

Click here to access/download;Figure;Fig5.tif.tif

Diagnosis	PE 1	PE 2	PE 3	AL 1	AA 1	MM	IgA	MN	ANCA	AL 2	AA 2
Protein concentration, mg/ml	1.5	5.0	4.8	0.8	5.3	2.4	2.8	4.7	2.1	4.7	4.9

CF, 4 000 x*g*, 10 min
CRR, **0.90**, C.I., 0.759–1.029

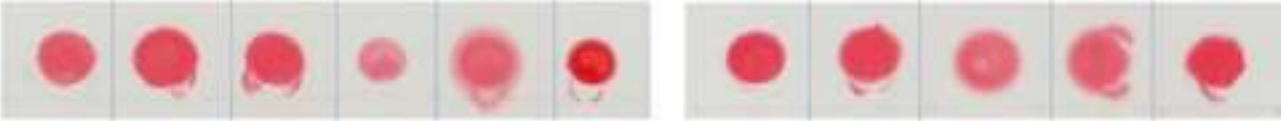

CF, 300 000 x*g*, 120 min
CRR, **0.98**, C.I., 0.853–1.109

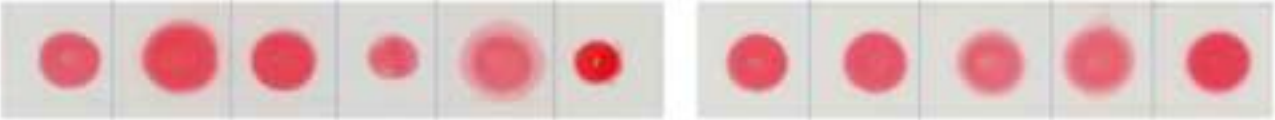

Figure 6

Click here to access/download;Figure;Fig6.tif.tif

A

Diagnosis	PE	AL	HSA
Protein concentration, mg/ml	5.0	0.8	2.0
Original sample			
<30 kDa MW (filtrate)			
>30 kDa MW (concentrate)			
<100 kDa MW (filtrate)			
>100 kDa MW (concentrate)			

B

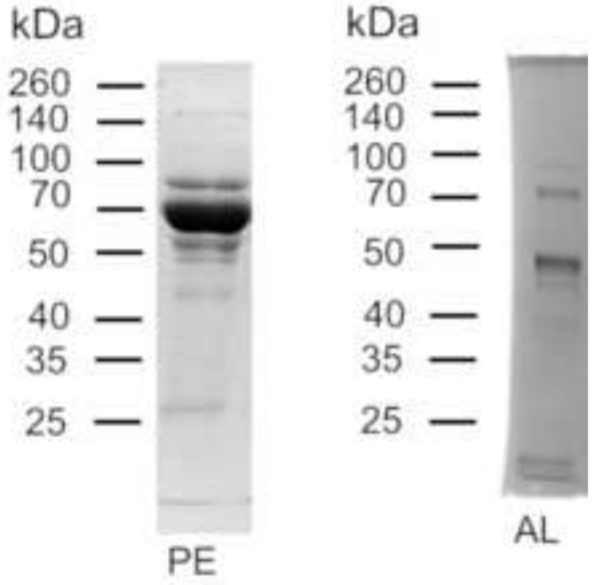

Figure 7 Click here to access/download;Figure;Fig7.tif.tif

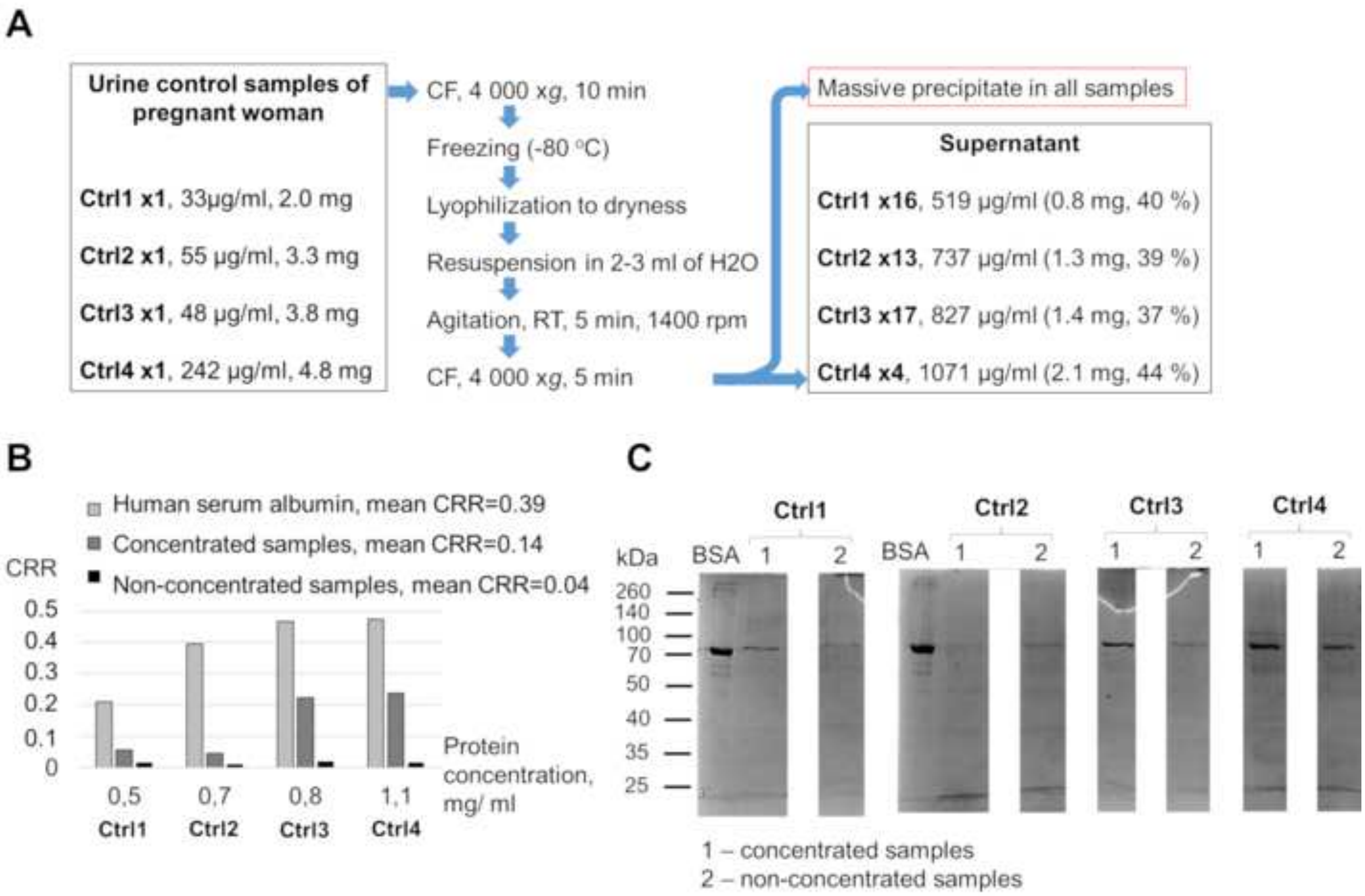

Supplement: Attachment — Submitted filename: PONE-D-23-29876 (2).pdf [file pone.0297144.s003.pdf]
